# ^18^F-Trifluoromethylated D-Cysteine as a Promising New PET Tracer for Glioma Imaging: Comparative Analysis With MRI and Histopathology in Orthotopic C6 Models

**DOI:** 10.3389/fonc.2021.645162

**Published:** 2021-04-29

**Authors:** Hui Ma, Jing Zhao, Shaoyu Liu, Dingxiang Xie, Zhanwen Zhang, Dahong Nie, Fuhua Wen, Zhiyun Yang, Ganghua Tang

**Affiliations:** ^1^Department of Radiology, The First Affiliated Hospital, Sun Yat-sen University, Guangzhou, China; ^2^Department of Nuclear Medicine, Guangdong Engineering Research Center for Translational Application of Medical Radiopharmaceuticals, The First Affiliated Hospital, Sun Yat-sen University, Guangzhou, China; ^3^Department of Nuclear Medicine, The First Affiliated Hospital of Guangzhou Medical University, Guangzhou, China; ^4^Department of Nuclear Medicine, The Sixth Affiliated Hospital, Sun Yat-sen University, Guangzhou, China; ^5^Department of Radiation Oncology, The First Affiliated Hospital, Sun Yat-sen University, Guangzhou, China; ^6^Nanfang PET Center, Department of Nuclear Medicine, Nanfang Hospital, Southern Medical University, Guangzhou, China

**Keywords:** glioma, PET imaging, MRI, PET tracers, amino acid

## Abstract

Comparing MRI and histopathology, this study aims to comprehensively explore the potential application of ^18^F-trifluoromethylated D-cysteine (*S-*[^18^F]CF_3_-D-CYS) in evaluating glioma by using orthotopic C6 glioma models. Sprague–Dawley (SD) rats (*n* = 9) were implanted with C6 glioma cells. Tumor growth was monitored every week by multiparameter MRI [including dynamic contrast-enhanced MRI (DCE-MRI)], [^18^F]FDG, *S-*[^18^F]CF_3_-D-CYS, and [^18^F]FDOPA PET imaging. Repeated scans of the same rat with the two or three [^18^F]-labeled radiotracers were investigated. Initial regions of interest were manually delineated on T_2_WI and set on the same level of PET images, and tumor-to-normal brain uptake ratios (TNRs) were calculated to semiquantitatively assess the tracer accumulation in the tumor. The tumor volume in PET and histopathology was calculated. HE and Ki67 immunohistochemical staining were further performed. The correlations between the uptake of *S-*[^18^F]CF_3_-D-CYS and Ki67 were analyzed. Dynamic *S-*[^18^F]CF_3_-D-CYS PET imaging showed tumor uptake rapidly reached a peak, maintained plateau during 10–30 min after injection, then decreased slowly. Compared with [^18^F]FDG and [^18^F]FDOPA PET imaging, *S-*[^18^F]CF_3_-D-CYS PET demonstrated the highest TNRs (*P* < 0.05). There were no significant differences in the tumor volume measured on *S-*[^18^F]CF_3_-D-CYS PET or HE specimen. Furthermore, our results showed that the uptake of *S-*[^18^F]CF_3_-D-CYS was significantly positively correlated with tumor Ki67, and the poor accumulated *S-*[^18^F]CF_3_-D-CYS was consistent with tumor hemorrhage. There was no significant correlation between the *S-*[^18^F]CF_3_-D-CYS uptakes and the K^trans^ values derived from DCE-MRI. In comparison with MRI and histopathology, *S-*[^18^F]CF_3_-D-CYS PET performs well in the diagnosis and evaluation of glioma. *S-*[^18^F]CF_3_-D-CYS PET may serve as a valuable tool in the clinical management of gliomas.

## Introduction

Gliomas are the most common primary intracranial tumor with high mortality and poor prognosis. The high tumor heterogeneity and invasiveness present a considerable obstacle to the treatment of glioma, which was the reason for causing tumor recurrence and treatment resistance (1). Thus, seeking a non-invasive modality to the full extent of depicting the tumor invasion and demonstrating the tumor heterogeneity is proficient for glioma diagnosis and treatment design. MRI has been routinely and wildly used to evaluate gliomas. However, the conventional and advanced MRI sequences are still not good enough to identify the tumor boundary and to provide all needed pathophysiological information of gliomas (2, 3). Beyond the MRI, PET provides additional insights into the pathophysiology of gliomas (4, 5).

[^18^F]FDG is the most commonly used PET tracer in the clinic, and it has been widely used to evaluate a variety of cancers, including gliomas. However, the high uptakes of [^18^F]FDG in both tumor and normal gray matter bring a strong challenge for the diagnosis of gliomas and tumor boundary delineation. Currently, amino acid (AA) tracers have been used predominantly for glioma imaging and exhibit lower uptake in a normal brain tissue, which is better suitable for the delineation of tumor extent and treatment planning than [^18^F]FDG (6). This would be explained by the fact that, compared with the normal tissue, the AA transporters such as LAT-1 (belonging to system L) are overexpressed in gliomas (7, 8). Among all types of amino acid tracers, *S-*[^11^C]methyl-L-methionine ([^11^C]-MET) is preferred in clinical use (9, 10). Some investigations have demonstrated that [^11^C]-MET had a higher sensitivity and a lower specificity, which varied between 75 and 100%. Unfortunately, [^11^C]-MET is not an ideal tumor tracer since inflammatory processes are also known to show increased [^11^C]-MET uptake, and the short half-life of the radionuclide ^11^C (20.38 min) further limits the extensive clinical application of [^11^C]-MET (11). In addition, 6-[^18^F]fluoro-L-3,4-dihydroxyphenylalanine ([^18^F]FDOPA, a classic dopamine neurotransmitter imaging agent) has recently been used for evaluating glioma and demonstrated promising results in predicting low-grade glioma prognosis and in diagnosing recurrent glioma (12, 13). However, it cannot be ignored that the high uptake in basal ganglia of [^18^F]FDOPA may have an impact on the delineation of glioma boundary. Besides, the longer half-lives (109.7 min) and similar transport mechanism to [^11^C]-MET make O-(2-[^18^F]fluoroethyl)-L-tyrosine ([^18^F]-FET, a tyrosine analog radiolabeled PET tracer) more suitable for clinical use (14). However, the slow renal elimination and some false negatives in diagnosing gliomas make [^18^F]-FET flawed (15). Thus, novel PET tracers for good imaging of gliomas are still in demand.

Taking the advantage of ^18^F with a relatively long half-life time (109.7 min) into consideration, our group has developed a pair of novel ^18^F-labeled AA PET tracers (^18^F-trifluoromethylated D- and L-cysteines) (16). They were designed as “structure-mimetic” amino acid tracers via replacement of methyl group with the ^18^F-trifluoromethyl group according to a structure-based bioisosterism strategy. Based on a series of cellular and biological evaluations, our preliminary results suggested that ^18^F-trifluoromethylated D-cysteine (*S-*[^18^F]CF_3_-D-CYS) was a promising PET tracer for evaluating gliomas. In addition, *S-*[^18^F]CF_3_-D-CYS is an analog of *S-*[^11^C]methyl-L-cysteine, in which its low uptake by the brain tissue has been reported and was able to detect recurrent glioma (17). Hence, in this study, we plan to further evaluate the ability of *S-*[^18^F]CF_3_-D-CYS PET imaging in the diagnosis of glioma compared to [^18^F]FDG and [^18^F]FDOPA PET with reference to MRI and histopathology through orthotropic C6 glioma models.

## Materials and Methods

### Study Design

This study has complied with the recommendations of the Guidance for the Care and Use of Laboratory Animals of the Ministry of Science and Technology of the People's Republic of China. Our experiments were approved by the Institutional Animal Care and Use Committee (IACUC), Sun Yat-sen University (Permit Number SYSU-IACUC-2019-000057).

**Figure 1:**
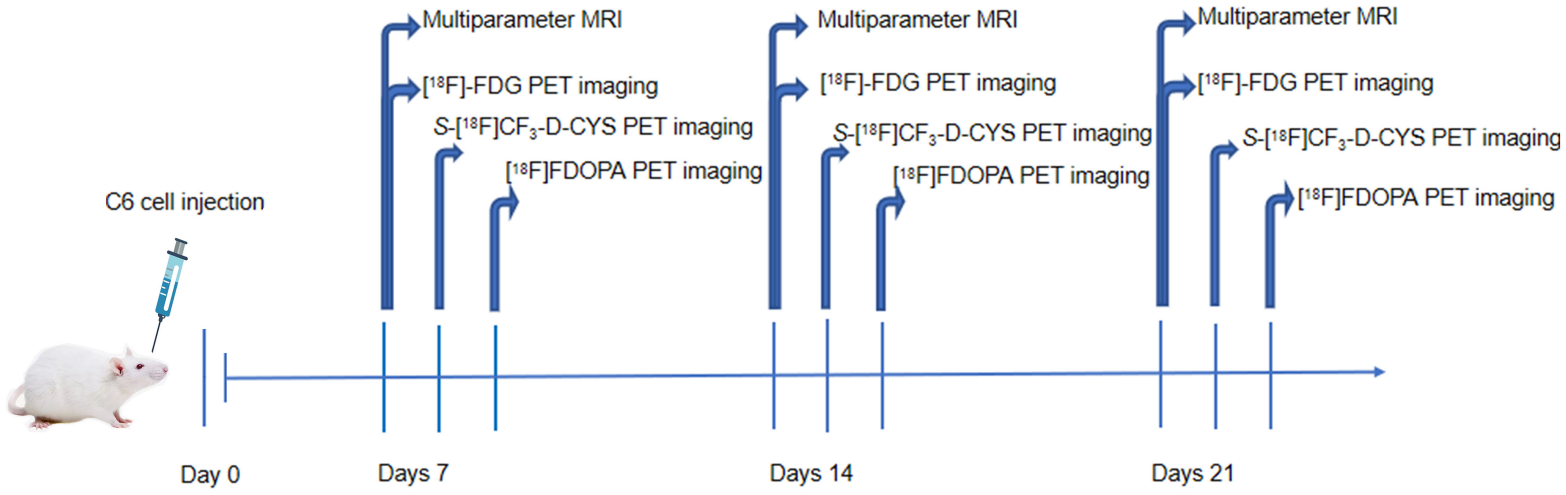
The outline of this study.

The male Sprague–Dawley (SD) rats (*n* = 9) were stereotactically injected with C6 glioma cells for the establishment of orthotropic C6 glioma models. Multiparameter MRI including conventional MRI and DCE-MRI and [^18^F]FDG PET imaging was performed on the 7th, 14th, and 21st days after the operation. Subsequently, *S-*[^18^F]CF_3_-D-CYS and [^18^F]FDOPA PET imaging was performed in the succeeding days. Experimental procedures are shown in Figure 1. After imaging acquisition, tumor-bearing rat models were sacrificed for pathological examinations including hematoxylin–eosin staining (HE) and Ki67 immunohistochemical staining. During the whole experimental procedures, if the rats were dead (glioma-bearing rats were vulnerable to death), they were immediately subjected to pathological examinations.

### Cell Culture and Animal Models

C6 glioma cells were purchased from the Cell Bank of the Chinese Academy of Sciences in Shanghai. Cells were cultivated in culture medium composed of 90% Dulbecco's modified Eagle's medium (GIBCO) and 10% fetal bovine serum (GIBCO) in a cell incubator with a humidified atmosphere of 5% CO_2_ and 95% air at 37°C. Cells were harvested or passaged with trypsinization when they reached over 90% confluence in the logarithmic growth phase.

To establish orthotropic C6 glioma models, about 1 × 10^6^ C6 cells in 10 μL of phosphate-buffered saline (PBS) was injected into the right hemisphere (the right caudate nucleus in more specific terms) of each male SD rat (6–8-weeks old, 200–250 g in weight) through a small-animal stereotactic instrument. During the operation, rats were in effective anesthetic condition with 2% pentobarbital (40 mg/kg), and the heads of rats were immobilized. Before cell injections, the skin of the heads was sterilized, and then, skulls were exposed with incisions. Through a burr hole placed 1 mm anteriorly and 3 mm right laterally to the bregma, cells in a 10-μL microsyringe were slowly injected into the white matter at a depth of 5 mm during 10 min. After 5–10 min pace times, the microsyringe was drawn, and the drilled hole was sealed. Furthermore, the wound was sutured, and animals were sent back to a specific pathogen-free laboratory animal room.

### Micro-PET/CT Protocol and Data Postprocessing

The preparation and radiosynthesis of *S-*[^18^F]CF_3_-D-CYS (16), [^18^F]FDG (18), and [^18^F]FDOPA (19, 20) were performed as previously described. PET/CT imaging was performed by an Inveon small-animal PET/CT scanner (Siemens, Knoxville). The rats were anesthetized with 2% pentobarbital (35 mg/kg), and they were intravenously injected with different PET tracers (100 μCi/kg, 37 MBq/kg) in 100–300 μL saline via tail veins. A low-dose CT scan was started followed by a PET scan. Following our study intentions, the dynamic and/or static data were acquired for *S-*[^18^F]CF_3_-D-CYS PET imaging, and an only static scan was acquired with [^18^F]FDG and [^18^F]FDOPA PET imaging. The 120-min dynamic acquisition of *S-*[^18^F]CF_3_-D-CYS PET was intended to depict the uptake curve of *S-*[^18^F]CF_3_-D-CYS in tumors and determin the optimal acquisition time point of *S-*[^18^F]CF_3_-D-CYS. For static acquisition, a 15-min static PET scan was performed for [^18^F]FDOPA at 10–30 min after injection and at 60 min after administration of [^18^F]FDG.

Two-dimensional ordered-subsets expectation maximum was used during image reconstruction. The 120-min dynamic *S-*[^18^F]CF_3_-D-CYS PET imaging was reconstructed every 1 min with 30 frames for the first 30 min and then every 5 min with 18 frames for the last 90 min. Using the Inveon Research Workplace 4.1 software, regions of interest (ROIs) of 2-mm diameter were drawn over tumor tissues and normal brain tissues (contralateral normal cerebral tissue excluding ventricles) of certain PET/CT images. Furthermore, radioactivity uptake of tissues was presented as mean % ID/g (the average uptake value of the three different ROIs over the same area). % ID/g means a percentage of the injected dose per gram of tissue.

### MRI Protocol

#### Conventional MRI

Before the experiment, the rats were fasted for 4 h and anesthetized with 2% pentobarbital (35 mg/kg). Furthermore, an intravenous needle was placed in the rats' tail vein for the contrast agent injection. Brain MRI was performed using a 3T MR system (Magnetom Verio, Siemens Medical Solutions, Erlangen, Germany) with eight phased-array animal coils. Transversal T_2_-weighted images [repetition time (TR), 4,800 ms; echo time (TE), 116 ms; field of view (FOV), 60 mm × 60 mm; slice thickness, 1.5 mm; voxel resolution, 0.2 mm × 0.2 mm × 1.5 mm), transversal T1-weighted images (TR, 660 ms; TE, 18 ms; FOV, 60 mm × 60 mm; slice thickness, 1.5 mm; voxel resolution, 0.3 mm × 0.2 mm × 1.5 mm), and susceptibility weighted imaging (SWI) (TR, 28 ms; TE, 20 ms; FOV, 60 mm × 60 mm; slice thickness, 0.75 mm; voxel resolution, 0.2 mm × 0.2 mm × 0.8 mm) were obtained. Postcontrast sagittal 3D T_1_-weighted images (TR, 1,990 ms; TE, 3.45 ms; section thickness, 0.35 mm; FOV, 90 mm × 90 mm; voxel resolution, 0.4 mm × 0.4 mm × 0.3 mm) were obtained after DCE-MRI.

#### DCE-MRI

T_1_-VIBE was applied at two different flip angles (2 and 15°) to calculate the T_1_ maps. Below were the parameters (TR, 7.07 ms; TE, 2.44 ms; slice thickness, 1.5 mm; FOV, 60 mm × 60 mm; voxel resolution, 0.5 mm × 0.4 mm × 1.5 mm). DCE-MRI (dynamic contrast-enhanced MR perfusion) was acquired with time-resolved angiography with stochastic trajectories (TWIST) sequence; the parameters were the following: TR, 6.27 ms; TE, 2.7 ms; flip angle, 12°; slice thickness, 1.5 mm; for each measurement, 3.6 s; FOV, 60 mm × 60 mm; 75 measurements, total scan time of 276 s; voxel resolution, 0.4 mm × 0.3 mm × 1.5 mm; contrast media (0.1 mmol/kg body weight of Gd-DTPA, Magnevist, Schering, Berlin, Germany); contrast median injection rate, 1 mL/s, followed by 20 mL of 0.9% saline flush using the same injection speed. Infusion started from the fifth measurement.

All DCE-MRI data were transferred to the postprocessing workstation. The analysis was done by a commercial software tool (TISSUE 4D; Siemens Healthcare, Erlangen, Germany). A value for the arterial input function was automatically calculated using the software. TISSUE 4D was based on the two-compartment model (21), and volume transfer constant (K^trans^) maps were automatically generated. In a line with the ROI placement in PET study, 2-mm diameter ROIs were drawn over tumor tissues and normal brain tissues.

### Pathological and Immunohistochemical Analysis

The whole rat brains were obtained and put in a 4% formalin fixation overnight. Then, they were cut into continuous 3-μm slices. Several stainings were further performed, and the methods were reported previously (22–24). The HE staining was performed to validate the tumor morphology and delineate the tumor boundary. The tumor length, width, and height were measured via the HE-stained pathological specimen. The tumor growth was assumed in an ellipsoidal way (25), and the tumoral volume was calculated by the following formulas: V = π/6 × length × width × height.

Immunohistochemical staining for Ki67 (monoclonal antibody, Servicebio, Wuhan, China, GB13030-2) was performed using the Envision method (22), and the Ki67 index represents the proliferative activity of glioma cells. The tumor sections were reviewed and quantified based on the percentage of positive cells in the highest density staining area; all cells with nuclear staining of any intensity were considered positive, and the Ki67 values were defined as the percentage of positive cells among the total cells counted (24).

### Image Coregistration

For each model, PET images were automatically coregistered to their paired CT images because of the same anatomical position during scanning. T_2_WI-MRI images of the same model were used as the reference of MRI images. Pathological images of the same model were considered as on the same level owing to the too narrow distance using the method of serial sections (3 μm), and HE images were used as the reference. Besides, images of different modalities were manually coregistered to each other among PET, MRI, and pathological images according to the anatomical structure.

### Statistical Analysis

Data were reported as mean ± standard deviation of the mean. Statistical analysis was performed with GraphPad Prism 8. Paired or unpaired Student's *t* test and one-way ANOVA tests were carried out to evaluate the significant differences among two or more groups. If each of the global F was significant (*P* < 0.05), Bonferroni analysis was used to assess the difference between single groups, according to the corresponding multiplicity-adjusted *P*-values. Pearson correlation analyses were performed to assess the correlation between the values of two groups. A *P* < 0.05 was statistically considered significant.

## Results

### The Optimal Imaging Time Point Acquisition for *S-*[^18^F]CF_3_-D-CYS PET Imaging

**Figure 2:**
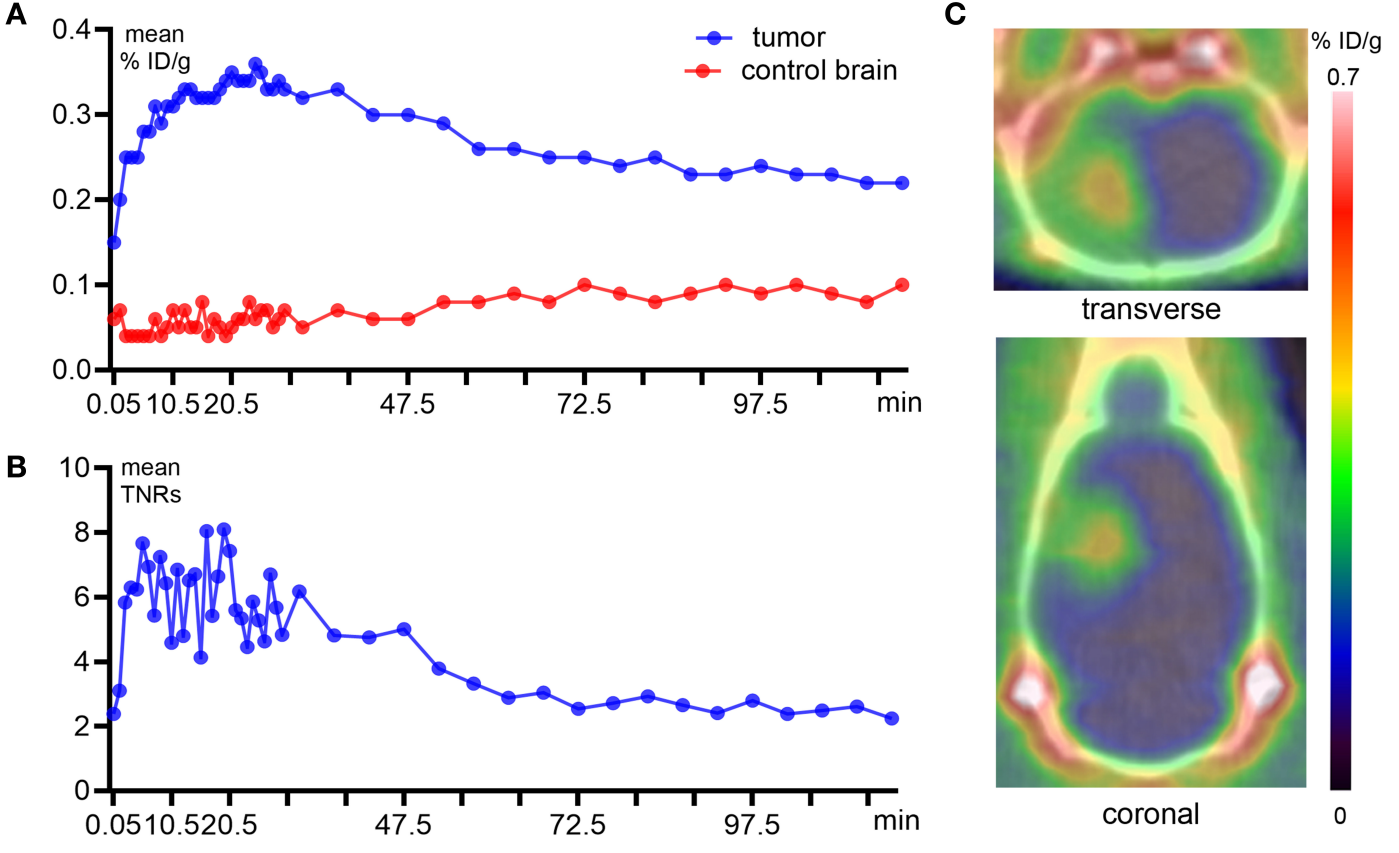
Dynamic PET imaging with *S-*[^18^F]CF_3_-D-CYS and quantitative analysis derived from a C6 orthotopic tumor model. **(A)** Quantitative analysis of the tumor and control brain uptake during 2 h after *S-*[^18^F]CF_3_-D-CYS administration. **(B)** The time–activity curves of uptake ratios of the tumor to normal brain. **(C)** Representative static *S-*[^18^F]CF_3_-D-CYS PET imaging, which was performed at 20 min after injection, and compared with the normal brain tissue, the tumor (red arrow) had higher uptake of *S-*[^18^F]CF_3_-D-CYS (right arrows: tumors).

The representative time–activity curves (TACs, Figure 2A) showed relatively high tumor uptake and low normal brain uptake. *S-*[^18^F]CF_3_-D-CYS reached the maximum accumulation in the tumors at 10 min after injection and with long-term retention; then, it began to slowly decline at 30 min after injection. Until postinjection of 2 h, the uptake of *S-*[^18^F]CF_3_-D-CYS in tumors was still higher than that of the normal brain. The mean uptake ratios of the TNRs are shown in Figure 2B, which was consistent with the abovementioned tendency of tumor uptake.

The dynamic TNR curve revealed that the mean TNRs of *S-*[^18^F]CF_3_-D-CYS were constant from 10 to 30 min p.i., which indicated that the optimal acquisition time for static PET imaging was at 10–30 min after injections. Thus, the frames during the above time period were summed to obtain static images (Figure 2C) that were used for good visualization of tumors.

### Assessment of the Efficiency of *S-*[^18^F]CF_3_-D-CYS PET Imaging in the Diagnosis of Gliomas in Terms of Tumor Boundary

#### The Highest Uptake TNRs and Early Tumor Detection

**Figure 3:**
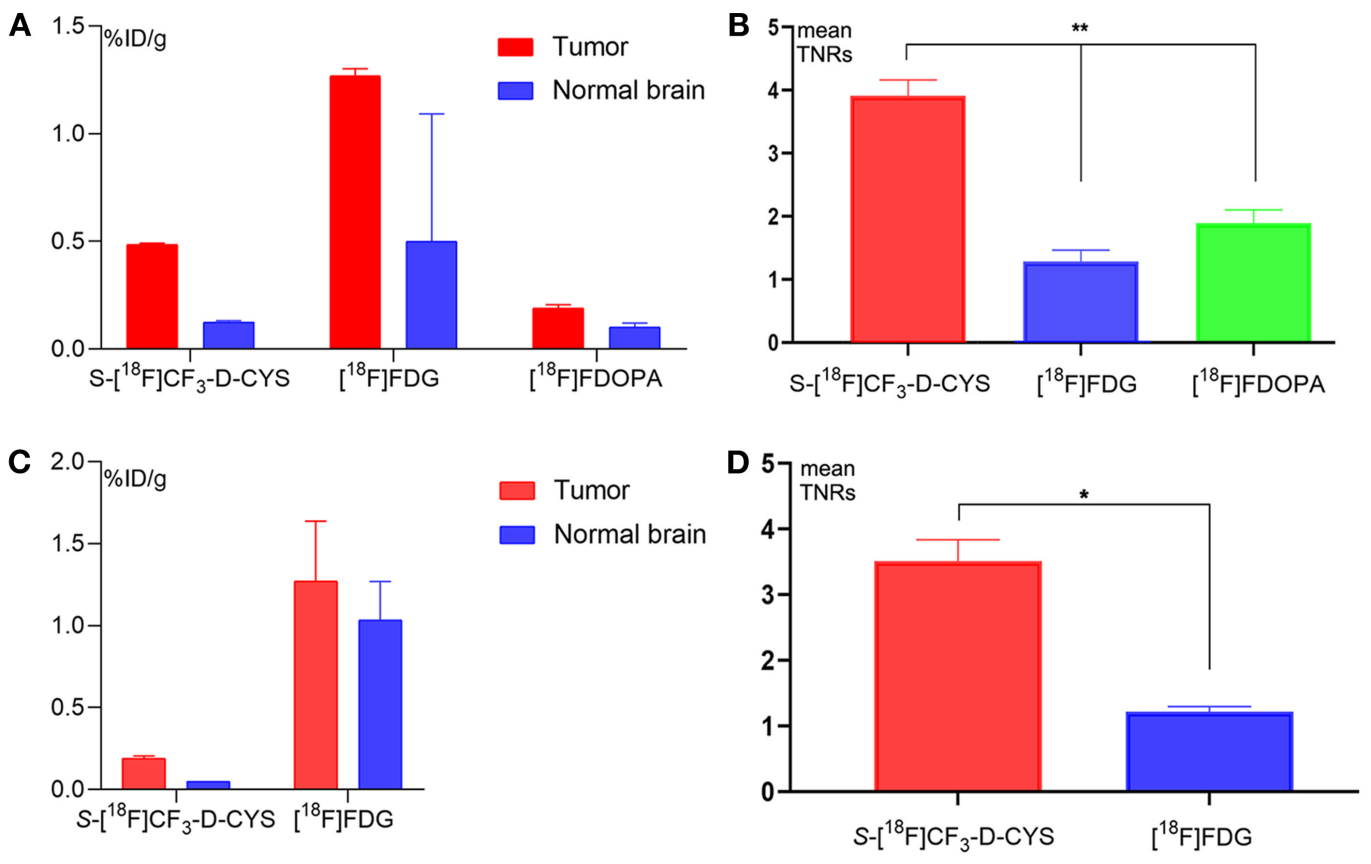
**(A,B)** The uptake values of tumors and normal brains **(A)**, and the mean uptake ratios of the tumor to normal brain **(B)** for PET imaging with *S-*[^18^F]CF_3_-D-CYS, [^18^F]FDG, and [^18^F]FDOPA for rats bearing orthotopic C6 glioma 3 weeks after xenografts (**P < 0.01). **(C,D)** The uptake values of tumors and normal brains **(C)** and the mean tumor and control brain uptake **(D)** for PET imaging with *S-*[^18^F]CF_3_-D-CYS and [^18^F]FDG for rats bearing orthotopic C6 glioma 1 week after xenografts. The mean TNRs for *S-*[^18^F]CF_3_-D-CYS PET images (3.51 ± 0.33) were nearly three times higher than those for [^18^F]FDG PET images (1.22 ± 0.08) (*P < 0.05).

**Figure 4:**
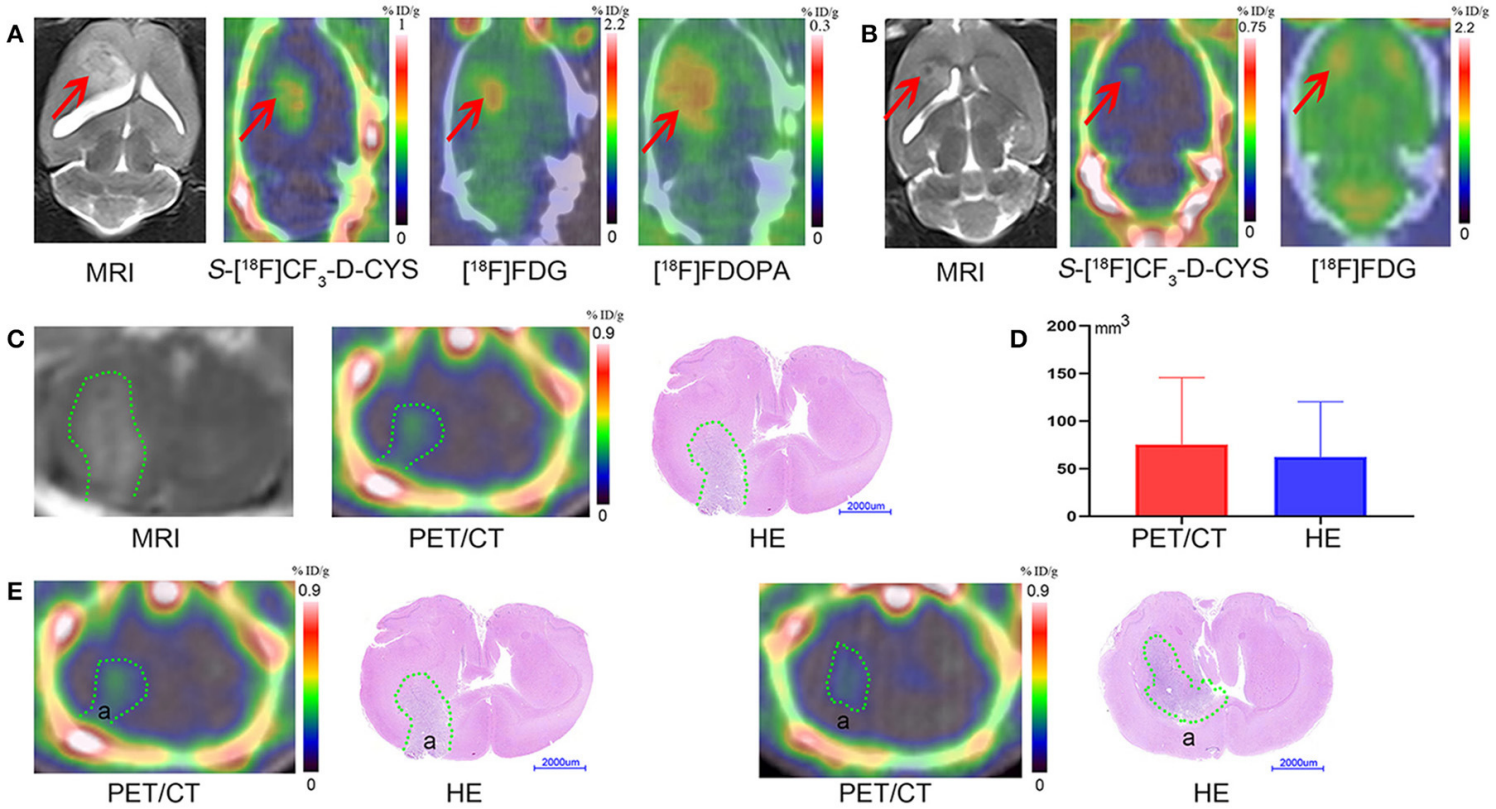
**(A)** The representative coronal images of the same rat model bearing orthotopic C6 glioma 3 weeks after xenografts with MR imaging, *S-*[^18^F]CF_3_-D-CYS PET imaging, [^18^F]FDG PET imaging, and [^18^F]FDOPA PET imaging (from left to right) (right arrows: tumors). **(B)** The representative coronal images of the same model rat bearing orthotopic C6 glioma 1 week after xenografts with MR imaging, *S-*[^18^F]CF_3_-D-CYS PET imaging, and [^18^F]FDG PET imaging (from left to right). Compared with MRI, the tumor with *S-*[^18^F]CF_3_-D-CYS PET imaging had a clear tumor boundary (red arrows: tumors). **(C)** Representative MRI images, *S-*[^18^F]CF_3_-D-CYS PET images, and whole-brain HE images on the parallel brain slices of the same rat model. **(D)** Comparison of the tumoral volume measured from *S-*[^18^F]CF_3_-D-CYS PET images and histopathology, showing that there was no significant difference (green curves: tumors). **(E)** Two successive slides of PET scan images and corresponding HE images. There was tumor infiltration in area a in the left images, while there was no tumor infiltration in area a in the right images.

The uptake values of tumors and normal brains of C6 gliomas with the same tumor age, under the PET examinations by using three PET tracers (*S-*[^18^F]CF_3_-D-CYS, [^18^F]FDG, and [^18^F]FDOPA) in the same rat were shown in Figure 3A. And the *S-*[^18^F]CF_3_-D-CYS PET imaging demonstrated the highest mean TNRs (Figure 3B). Taking the 3-week C6 glioma models as an example, *S-*[^18^F]CF_3_-D-CYS was significantly accumulated in the tumor area, where T_2_WI MRI showed uneven high intensity (Figure 4A). The mean TNRs of *S-*[^18^F]CF_3_-D-CYS PET (3.90 ± 0.25) were the highest compared with those of [^18^F]FDG PET (1.28 ± 0.18) and [^18^F]FDOPA PET (1.90 ± 0.21) (Figure 3B). Furthermore, *S-*[^18^F]CF_3_-D-CYS PET imaging could detect the tumor in the early stage, and compared with [^18^F]FDG PET, it showed higher TNRs and more clearly depicted the tumor boundary (Figures 3C,D, 4B).

#### Delineating Tumor Boundary Effectively and Close to the Pathological Tumor Volume

We selected the same section of *S-*[^18^F]CF_3_-D-CYS PET imaging, MRI, and HE-staining tumor specimen of a representative rat model as an example, which is shown in Figure 4C. Visually, compared with HE-staining tumor specimen, *S-*[^18^F]CF_3_-D-CYS PET imaging showed high accordance with pathology depicting this C6 glioma with irregular tumor boundary (Figure 4C). Besides, compared two successive slides of PET scan images and corresponding HE images (Figure 4E), *S-*[^18^F]CF_3_-D-CYS PET imaging could reflect changes in the tumor infiltration area shown as area in Figure 4E. Thus, *S-*[^18^F]CF_3_-D-CYS PET imaging could identify the irregular tumor boundary and differentiate the tumor and the normal brain tissue. Furthermore, there were no significant differences in the measured tumor volume via *S-*[^18^F]CF_3_-D-CYS PET images and histopathology (*n* = 3, *P* > 0.05; Figure 4D).

### Assessment of the Efficiency of *S-*[^18^F]CF_3_-D-CYS PET Imaging in the Diagnosis of Gliomas in Terms of Tumor Heterogeneity

#### A Significant Positive Correlation of S-[^18^F]CF_3_-D-CYS Uptake and the Tumor Ki67 Labeling Index

**Figure 5:**
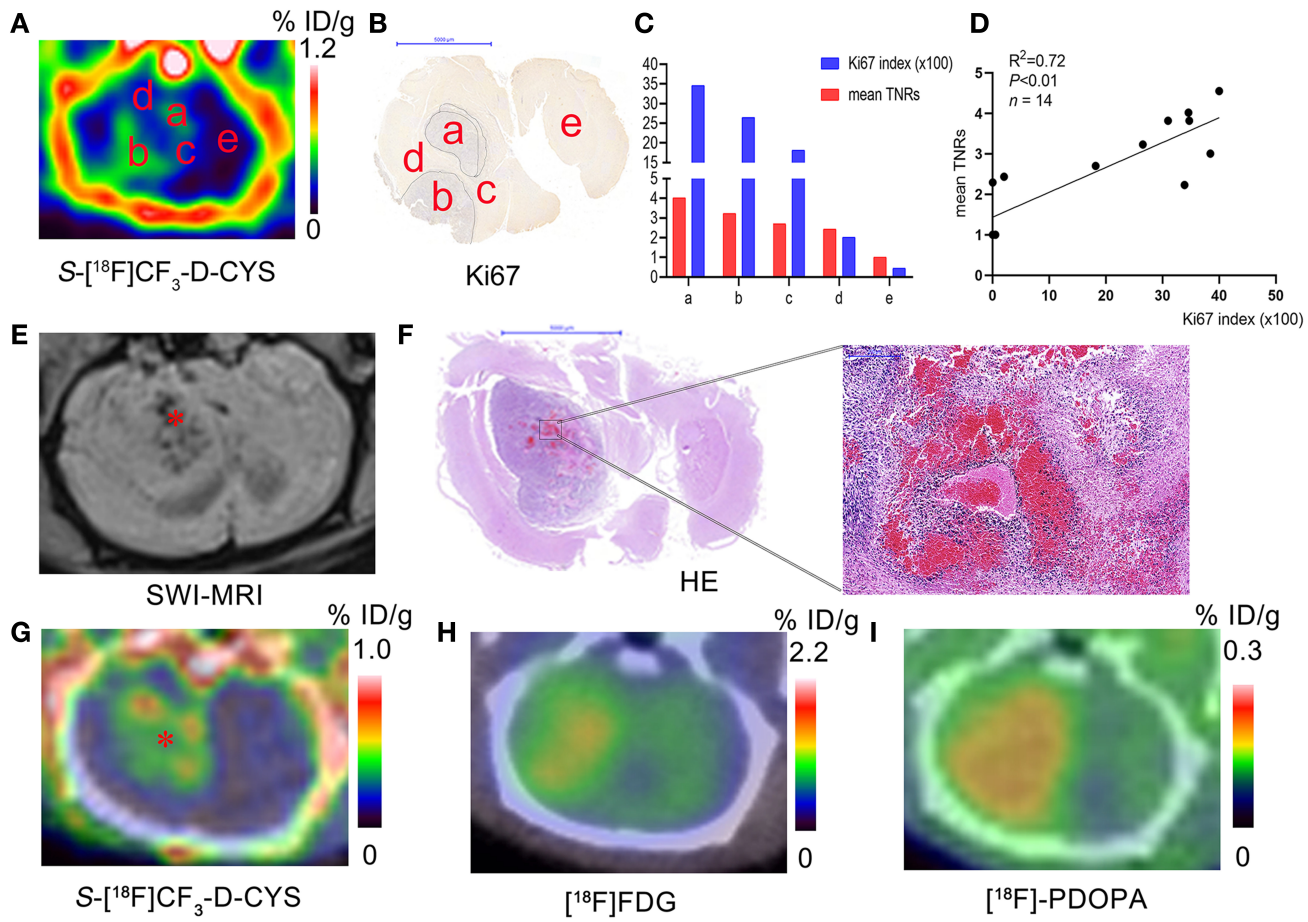
**(A–C)** Representative *S-*[^18^F]CF_3_-D-CYS PET images **(A)**, the corresponding Ki67 staining image **(B)**, and the corresponding uptake values and Ki67 index for regions a–e in Panel **A (C)** (a–e in Panels **A–C**: different tissue regions; tissue regions marked by the same letters in different corresponding images are considered as the same ones). **(D)** Correlation analysis between tumor *S-*[^18^F]CF_3_-D-CYS uptake values of different regions (such as marked tissue regions a–e in the Panel **A**) in several rat models (represented by mean TNRs) and the corresponding Ki67 labeling index. **(E–I)** The representative SWI-MRI image **(E)**, HE images of the whole-brain section and the high-magnification view **(F)**, *S-*[^18^F]CF_3_-D-CYS PET image **(G)**, [^18^F]FDG PET image **(H)**, and [^18^F]FDOPA PET image **(I)** on the corresponding section of the same C6 glioma model (* in **E**: the tumor region with blood; * in **G**: the tumor region with low *S-*[^18^F]CF_3_-D-CYS uptake).

As shown in Figure 5A, *S-*[^18^F]CF_3_-D-CYS uptake values were visually different in different regions such as areas a–e (pointed in Figures 5A–C) even in the same level of the C6 glioma. The mean Ki67 labeling index (34.57%) was generally higher in the PET strong-uptake area a than those (0.45%) in the PET uptake-negative area e (Figure 5C). Quantificationally, Figure 5D showed a significant positive correlation between tumor *S-*[^18^F]CF_3_-D-CYS uptake values (represented by mean TNRs) and the corresponding Ki67 labeling index (*n* = 14, *R*^2^ = 0.72, *P* < 0.01), indicating that tumor *S-*[^18^F]CF_3_-D-CYS uptake values could reflect the proliferative ability of tumor cells.

#### Low S-[^18^F]CF_3_-D-CYS Uptake in the Tumor Bleeding Areas

To further demonstrate the ability of *S-*[^18^F]CF_3_-D-CYS PET imaging in reflecting tumor heterogeneity, we performed SWI-MRI examinations to detect the tumor bleeding. Results showed that there was internal hemorrhage for some C6 gliomas, and the bleeding was further confirmed by HE results. Comparing the tumor hemorrhagic site in SWI-MRI and *S-*[^18^F]CF_3_-D-CYS PET images, we found visually low tumor uptakes of *S-*[^18^F]CF_3_-D-CYS in the bleeding region, as shown in the Figures 5E–I. Moreover, compared to the corresponding [^18^F]FDG (Figure 5H) and [^18^F]FDOPA (Figure 5I) PET images, the visual difference in tumor uptake in the tumor bleeding area was much more appealing for the corresponding *S-*[^18^F]CF_3_-D-CYS PET images (Figure 5G).

### Correlation With Blood Brain Barrier and Capillary Permeability

**Figure 6:**
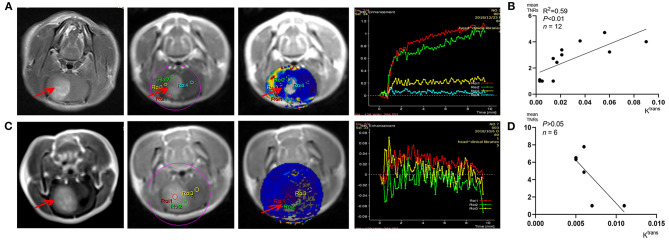
**(A,C)** Representative DCE-MRI analysis results and corresponding K^trans^ maps. **(B,D)** Correlation analysis results between K^trans^ values and corresponding tumor *S-*[^18^F]CF_3_-D-CYS uptakes (represented by mean TNRs) (right arrows: tumors).

All the tumors were vividly enhanced on T_1_ enhanced imaging, which indicated that C6 glioma did disrupt the blood–brain barrier (BBB). DCE-MRI analysis and concentration–time curve results (Figures 6A,C) showed that K^trans^ values in the tumors (such as ROIs 1–2 in Figure 6A) were higher than those of the contralateral normal brain parenchyma (such as ROI 4 in Figure 6A), indicating that C6 glioma had higher capillary permeability than the normal brain parenchyma. Figure 6B shows that the positive correlation between the *S-*[^18^F]CF_3_-D-CYS uptake values and the corresponding K^trans^ values in these tumor area (*n* = 12, *R*^2^ = 0.59, *P* < 0.01). However, there was an exception that there was no significant difference between the K^trans^ values of tumors and that of contralateral normal brain tissues (Figure 6C). Even so, the corresponding *S-*[^18^F]CF_3_-D-CYS uptakes of different ROIs in the tumor were still varied and higher than the contralateral normal parenchyma. Figure 6D showed that, in this kind of tumor, there was no significant correlation between *S-*[^18^F]CF_3_-D-CYS uptakes and K^trans^ values (*n* = 6, *P* > 0.05). Thus, the correlations between *S-*[^18^F]CF_3_-D-CYS uptakes and K^trans^ values were still debatable, and the tumor uptake of *S-*[^18^F]CF_3_-D-CYS was not necessary to depend on the degree of capillary permeability.

## Discussion

In this study, we successfully further explored and evaluated the application of a new sulfur-containing amino acid PET tracer (*S-*[^18^F]CF_3_-D-CYS) for evaluating glioma in orthotopic C6 glioma models. For *S-*[^18^F]CF_3_-D-CYS PET imaging, there was relatively high tumor uptake and low normal brain uptake. Compared with [^18^F]FDG and [^18^F]FDOPA PET imaging, *S-*[^18^F]CF_3_-D-CYS PET imaging had the highest TNRs, which was an advantage for tumor boundary delineation. The tumor volume in *S-*[^18^F]CF_3_-D-CYS PET imaging was close to the pathological size. Besides, we proved that *S-*[^18^F]CF_3_-D-CYS PET imaging could reflect the tumor hemorrhage and was significantly correlated with tumor cell proliferation. Furthermore, our results suggested that the uptake of *S-*[^18^F]CF_3_-D-CYS was independent of capillary permeability.

The dynamic PET scan of the glioma showed that the optimal imaging period for *S-*[^18^F]CF_3_-D-CYS PET was 10–30 min after trace injection. In detail, the TACs demonstrated that the accumulation of *S-*[^18^F]CF_3_-D-CYS in brain tumors was quick (within 10 min) and lasted for a long period (for 2 h), which makes the PET acquisition time of *S-*[^18^F]CF_3_-D-CYS flexible. Furthermore, our study showed that the uptake of *S-*[^18^F]CF_3_-D-CYS in the normal brain was relatively low, and it provided a good imaging contrast. Compared with [^18^F]FDG and [^18^F]FDOPA PET imaging, *S-*[^18^F]CF_3_-D-CYS PET imaging had the highest mean TNRs; it was possible to clearly define the C6 glioma boundary (26). Besides, compared with [^18^F]FDG, our study showed that *S-*[^18^F]CF_3_-D-CYS PET imaging could detect tumors in a week after orthotopic xenograft and even could delineate the tumor contour. This was a valuable finding, which has, on the other side, proven that the *S-*[^18^F]CF_3_-D-CYS PET imaging had high sensitivity in detecting gliomas. In addition, the tumor volume measured on *S-*[^18^F]CF_3_-D-CYS PET had high accordance with the pathological findings. Thus, *S-*[^18^F]CF_3_-D-CYS PET might serve as a valuable modality for glioma boundary delineation.

It is undeniable that the accumulation of *S-*[^18^F]CF_3_-D-CYS in the tumor area was variable, in which we inferred that the uneven uptake of *S-*[^18^F]CF_3_-D-CYS might indicate or reflect the tumor heterogeneity. We verified our inference in two aspects. On the one hand, our results showed that there was a significant positive correlation between *S-*[^18^F]CF_3_-D-CYS uptakes and the Ki67 labeling index. The higher the *S-*[^18^F]CF_3_-D-CYS uptakes were, the higher the tumor cell proliferation was. These findings are consistent with previous reports that [^11^C]-MET uptake correlated with the proliferative Ki67 index (27–29). On the other hand, we found that the tumor hemorrhage region, which was confirmed by SWI-MRI and histopathology, had a pretty low *S-*[^18^F]CF_3_-D-CYS uptake. Tumor cell proliferation rate and tumor with hemorrhage were thought to be related to tumor heterogeneity (12, 13). As a result, *S-*[^18^F]CF_3_-D-CYS is a promising candidate for evaluating glioma and reflecting tumor heterogeneity.

Interestingly, we explored whether *S-*[^18^F]CF_3_-D-CYS accumulation is dependent on BBB breakdown and capillary permeability. K^trans^ is a measure of capillary permeability obtained using DCE-MR perfusion. The negative correlation between *S-*[^18^F]CF_3_-D-CYS uptake and K^trans^ values including all cases showed that *S-*[^18^F]CF_3_-D-CYS uptake is independent on the degree of capillary permeability. However, we noticed that when the tumor has high capillary permeability, the uptake of *S-*[^18^F]CF_3_-D-CYS uptake was significantly correlated to the extent of capillary permeability, which we speculated that the increased capillary permeability might increase passive diffusion, which would help *S-*[^18^F]CF_3_-D-CYS enter and accumulate in the tumor area. Those results are in line with earlier studies reporting that AA transport into brain tumors is not dependent on, but may be intensified by, breakdown in the BBB (30, 31). Consequently, *S-*[^18^F]CF_3_-D-CYS PET imaging has another advantage over MRI in imaging tumors with intact BBB.

Going even further, the expression of some AA transporters, particularly the system L subtype LAT-1, system ASC subtype ASCT-2, and so on, is increased in malignant lesions (32, 33). In addition, our previous work (16) has identified that the cellular uptake of *S*-[^18^F]CF_3_-D-CYS in C6 cells mainly relied on the systems L (without the presence of Na+) and ASC (in the presence of Na+). Thus, we reasonably deduce that LAT-1 can transport *S*-[^18^F]CF_3_-D-CYS in the absence of Na+, and ASCT-2 is likely one of the transporters for uptake of *S*-[^18^F]CF_3_-D-CYS *in vitro*. These two transporters may play some roles *in vivo* as well. However, the transport mechanism of *S*-[^18^F]CF_3_-D-CYS is not clear yet. The final and precise conclusions with the tumor uptake mechanism of *S-*[^18^F]CF_3_-D-CYS still need to be further studied. Western blot or mass spectrometry to quantify transporter proteins needs to be further explored.

There are some comparative advantages of *S*-[^18^F]CF_3_-D-CYS over common clinical PET agents in imaging glioma. First, as D-isomers of PET enantiopure tracers, *S*-[^18^F]CF_3_-D-CYS, familiar with previous studies such as 3-[^18^F]fluoro-a-methyl-D-tyrosine (D-[^18^F]FAMT) and (D)-^18^F-fluoromethyltyrosine (D-^18^F-FMT), has some properties compared with their corresponding L-amino acids (16, 34, 35). For instance, D-isomers are characterized by fast clearance from the blood and kidney, thus low radiation burden, since D-amino acids rarely are used for the biological activity of mammals and there is rare accumulation in normal tissues. In this study, unlike the high uptake of [^18^F]FDG in the normal brain, there is an extremely low uptake of *S*-[^18^F]CF_3_-D-CYS in the normal brain. Therefore, it is easier to detect early tumors and more distinct to delineate tumor boundary for *S*-[^18^F]CF_3_-D-CYS PET imaging due to higher TNRs. Second, in our study, compared with [^18^F]FDOPA, the TNRs of *S*-[^18^F]CF_3_-D-CYS are higher, which is more suitable for depicting the tumor boundaries. Moreover, unlike the physiological uptake of [^18^F]FDOPA in striatum that mimics the tumor infiltration (6), there is no such observation for *S*-[^18^F]CF_3_-D-CYS PET imaging. For example, as is shown in Figure 4A, the tumor size depicted by [^18^F]FDOPA was larger than that contoured by *S*-[^18^F]CF_3_-D-CYS. It is because the high uptake in the basal ganglia of [^18^F]FDOPA confused the boundary contour of gliomas transplanted in the caudate nucleus. Third, compared with [^18^F]-FET, *S*-[^18^F]CF_3_-D-CYS is more suitable for discriminating metabolically active tumors from the vessels because the [^18^F]-FET has a longer retention time in the blood pool than *S*-[^18^F]CF_3_-D-CYS (15). Fourth, *S*-[^18^F]CF_3_-D-CYS seems to have similar potential in differentiating between tumor and inflammation compared to ^18^F-FBPA (16, 36). However, the D-isomer of ^18^F-FBPA was unsuitable for tumor PET probe as recently reported by Hirai et al. (37). Lastly, contrary to 3′-deoxy-3′-[^18^F]fluorothymidine ([^18^F]-FLT, a thymine nucleoside radiotracer not an AA, which indicates the proliferative state of cells), whose uptake is facilitated by BBB breakdown (15, 38), the uptake of *S*-[^18^F]CF_3_-D-CYS is independent of BBB breakdown, which lead a more extensive imaging range of various gliomas for *S*-[^18^F]CF_3_-D-CYS PET imaging.

However, one of the limitations of this study is the lack of autoradiographic imaging that can provide the precise comparison with histology. There could also be slow defluorination or bone intake of *S-*[^18^F]CF_3_-D-CYS, which may influence image quality. Even so, the superiority of *S-*[^18^F]CF_3_-D-CYS PET imaging in conjunction with multiparametric MRI during the long process of struggling against glioma should not be overlooked. Additional investigations are warranted in the future to improve its stability *in vivo* and optimize image effect.

In summary, our study evaluated a new ^18^F-trifluoromethylated D-cysteine, *S-*[^18^F]CF_3_-D-CYS, as a promising brain tumor PET tracer referred from previous research. The *S-*[^18^F]CF_3_-D-CYS PET imaging exhibited high tumor uptake and clear tumor boundaries, which were the clearest compared with those that [^18^F]FDG and [^18^F]FDOPA PET imaging depicted in our study. The uptake of *S-*[^18^F]CF_3_-D-CYS reflected tumoral heterogeneity and was independent of the degree of capillary permeability. In conclusion, in comparison with multiparametric MRI and histopathology, the *S-*[^18^F]CF_3_-D-CYS PET provides extensive information and has an excellent effect on glioma biopsy or neurosurgical planning and tumor diagnosis.

## Data Availability Statement

The raw data supporting the conclusions of this article will be made available by the authors, without undue reservation.

## Ethics Statement

This study has complied with the recommendations of the Guidance for the Care and Use of Laboratory Animals of the Ministry of Science and Technology of the People's Republic of China. Our experiments were approved by the Institutional Animal Care and Use Committee (IACUC), Sun Yat-sen University (Permit Number: SYSU-IACUC-2019-000057).

## Author Contributions

HM, JZ, SL, and GT: design of the study. SL and FW: synthesis of tracers. HM, JZ, and ZZ: animal experiments. DX: image scanning. DN, ZY, and GT: supervision of experiments. HM: original manuscript writing. All authors: discussion and analysis of results, critical feedback, revisions, and approval of the manuscript.

## Conflict of Interest

The authors declare that the research was conducted in the absence of any commercial or financial relationships that could be construed as a potential conflict of interest.
