# Supplementary material for: 18F-Trifluoromethylated D-Cysteine as a Promising New PET Tracer for Glioma Imaging: Comparative Analysis With MRI and Histopathology in Orthotopic C6 Models
Source: Front Oncol. 2021 Apr 29;11:645162. doi: 10.3389/fonc.2021.645162 (PMC8117348; doi:10.3389/fonc.2021.645162)
Supplement: Supplementary file 1 [file Image_1.pdf]

Supplementary Material

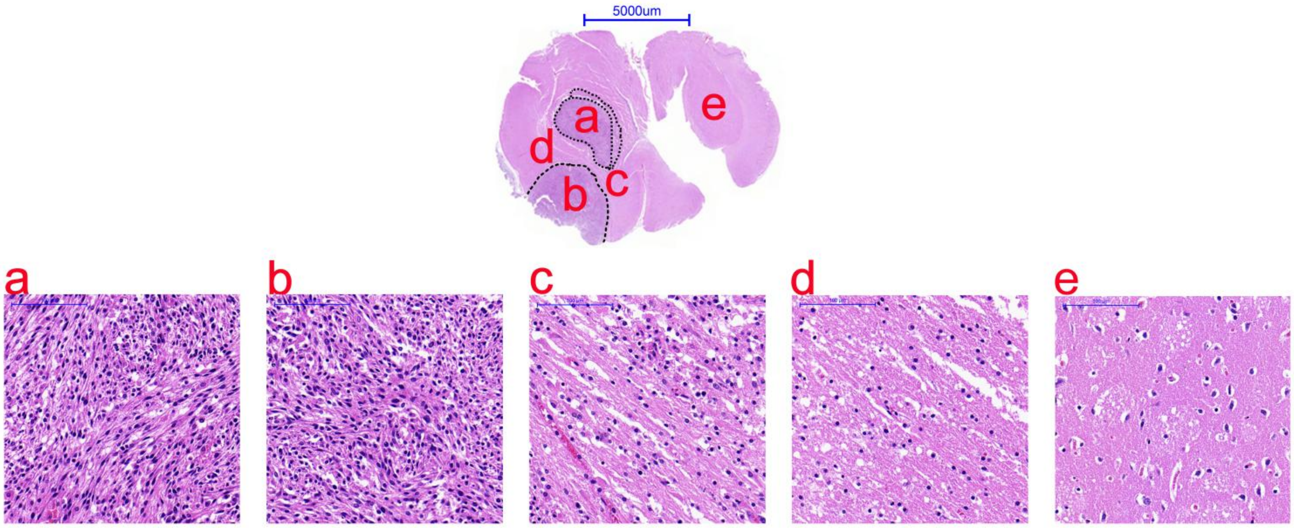

Supplementary Figure 1 HE images at high magnification of tissue regions marked by letters a, b, c, d, and e. (Notes: a and b were represented the tumors-infiltrating areas with dense cells; c and d were represented few tumor-infiltrating areas between a and b; e represented the normal brain.)
